# Phase-variable restriction/modification systems are required for *Helicobacter pylori* colonization

**DOI:** 10.1186/s13099-014-0035-z

**Published:** 2014-09-05

**Authors:** Jonathan C Gauntlett, Hans-Olof Nilsson, Alma Fulurija, Barry J Marshall, Mohammed Benghezal

**Affiliations:** 1 Ondek Pty Ltd and Helicobacter pylori Research Laboratory, School of Pathology and Laboratory Medicine, Marshall Centre for Infectious Disease Research and Training, University of Western Australia, Nedlands 6009, Western Australia, Australia

**Keywords:** Phase variation, Restriction, Modification, Phasevarion, Helicobacter pylori

## Abstract

**Background:**

One mechanism utilized by bacterial pathogens for host adaptation and immune evasion is the generation of phenotypic diversity by the phasevarion that results from the differential expression of a suite of genes regulated by the activity of a phase-variable methyltransferase within a restriction modification (RM) system. Phasevarions are active in *Helicobacter pylori,* however there have been no studies investigating the significance of phase-variable RM systems on host colonization.

**Methods:**

Two mutant types incapable of phase variation were constructed; a clean deletion mutant (‘DEL’) and a mutant (‘ON’) where the homopolymeric repeat was replaced with a non-repeat synonymous sequence, resulting in expression of the full-length protein. The resulting mutants were assessed for their colonisation ability in the mouse model.

**Results:**

Five phase-variable genes encoding either methyltransferases or members of RM systems were found in *H. pylori* OND79. Our mutants fell into three categories; 1, those with little effect on colonization, 2, those where expression of the full-length protein was detrimental, 3, those where both mutations were detrimental.

**Conclusions:**

Our results demonstrated that phase-variable methyltransferases are critical to *H. pylori* colonization, suggesting that genome methylation and generation of epigenetic diversity is important for colonization and pathogenesis. The third category of mutants suggests that differential genome methylation status of *H. pylori* cell populations, achieved by the phasevarion, is essential for host adaptation. Studies of phase-variable RM mutants falling in the two other categories, not strictly required for colonization, represent a future perspective to investigate the role of phasevarion in persistence of *H. pylori*.

## 1 Background

Phase variation is high-frequency, reversible ON/OFF switching of gene expression that results from slipped-strand mispairing at homopolymeric repeats present within alleles. Alteration of repeat length often leads to the insertion/removal of STOP codon within the phase variable gene [1]. This strategy is utilized by pathogenic bacteria as a mechanism to generate phenotypic diversity to adapt to their host [2]. Additional diversity is generated when the modification component (mod) of a restriction modification (RM) system phase varies. In RM systems the restriction (res) component cleaves unmethylated DNA at specific recognition sequences and the mod component methylates the same sequence to prevent restriction [3]. When this sequence occurs within a gene promoter, expression can be altered by the promoter’s differing methylation status [4]. Consequently, phase variation (ON/OFF) of a mod gene can lead to differential expression of a suite of genes, termed the phasevarion [5], enabling a population of cells to be differentiated based on multiple phenotypic characteristics [6].

*Helicobacter pylori* is a Gram-negative spiral-shaped bacterium that inhabits the human gastric mucosa of more than half of the world’s population [7] and is the causative agent of chronic gastritis and peptic ulcer. *H. pylori* has also been recognized as a risk factor for gastric cancer [8]. The *H. pylori* genome is replete with RM systems, with each strain containing its own unique complement [9], and its DNA is widely methylated by these systems [10].

We have previously reviewed the occurrence of phase variable genes and their role in generating phenotypic diversity in *H. pylori*[11]. Studies have identified potential phase-variable genes in *H. pylori*, including members of RM systems, and experimental evidence exists to demonstrate the presence of functional phasevarions [6],[12],[13]. However, there have been few studies demonstrating the significance of the phasevarion for colonization, persistence and adaptation *in vivo*. In one study, changes in the repeat length of 31 phase variable genes following murine gastric colonization were determined. Whilst changes were noted in many genes, indicating a role for phase variation in persistence, no temporal pattern could be determined for phase-varying restriction modification genes [14]. Here we construct a series of mutants in RM systems of *H. pylori* OND79 in order to investigate the significance of phase-variable RM systems on host colonization.

## 2 Results and discussion

Previous analysis of *H. pylori* genomes revealed 46 candidate phase-variable genes, with nine in the RM category [12]. We confirmed the presence of six of these genes in the genome of *H. pylori* strain OND79 (Table 1). The first gene, HP0585, is not a member of an RM system and does not contain homopolymeric repeats common to phase variable genes.

**Table 1:** **Presence of predicted phase variable DNA restriction/modification genes identified by Salaün** ***et al*. [**[12]**] in the genome of OND79**

**ORF**	**26695 ORF**	**Present in OND79**	**OND79 phase**	**Repeat**
Nth (endonuclease III)	HP0585	Yes	On	none
Adenine-specific methyltransferase	HP1353-4	No		
Type III RM system M protein	HP1369-70	Yes	Off	
Type II RM system β-subunit	HP1471	Yes	Off	G13
Type II RM system M protein	HP1522	Yes	Off	G10
HsdR (type I restriction enzyme)	HP0464	Yes	Off	C9
Cytosine-specific methyltransferase	HP0051	No		
MboIIR (type II restriction protein)	HP1366	No		G9
HemK or HemG (methyltransferase)	HP0381	Yes	On	G7, G7

**Figure 1:**
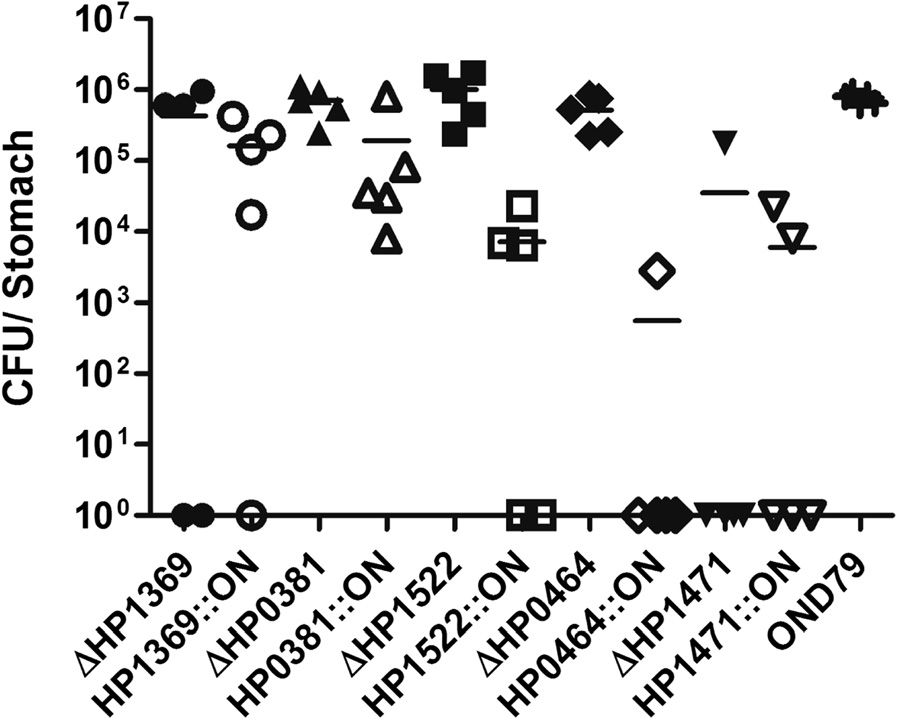
***H. pylori*and isogenic mutants were used to inoculate five eight-week old C57BL/6 J mice.** 2 weeks after challenge colonisation levels in stomachs were measured based on colony forming unit (CFU). Data is presented as a scatter plot with each point representing the CFU count of one mouse stomach, and the solid line the geometric mean for each group. Clean deletion mutants (filled symbols); homopolymeric repeat mutants (open symbols).

The remaining five genes were either methyltransferases or members of RM systems. For each gene we constructed both a clean deletion mutant (‘DEL’) and a mutant (‘ON’) where the homopolymeric repeat was replaced with a non-repeat synonymous sequence. This corrected the reading frame for expression of the full-length protein. Both mutant types are hence incapable of phase variation; one mutant is incapable of expressing the gene and the other produces only the full-length protein. Of note, none of the mutations in the 5 RM systems led to a growth defect of the corresponding mutants compared to wild-type, as observed during repeated passage on blood agar Petri dishes. Mice were orally challenged with the mutant strains and the bacterial load was determined following two weeks colonization (Figure 1).

Mutation of two genes, HP0381 and HP1369, had little effect on mouse colonization. HP0381 belongs to the HemK family of methyltransferases that methylate glutamine residues on release factors required for termination of translation and does not methylate DNA [15]. The type III adenine-specific methyltransferase, HP1369, is well conserved across sequenced *H. pylori* strains and correction of the frame-shift in the repeat has been shown to activate the enzyme [10]. The function of HP1369 does not appear to be significant for murine colonization. It is possible that correction of the frame-shift in HP1369 does not restore expression nor function in OND79.

Replacement of the repeat in HP1522 and HP0464 had a detrimental effect on the ability of OND79 to colonize mice. In *H. pylori* strain P12, deletion of the *modH5* allele (HP1522) led to differential regulation of six genes with the adhesin, HopG, significantly upregulated [6]. The expression of HP1522 in OND79 may reduce levels of HopG, resulting in the observed colonization defect. The res encoded by HP0464 is a member of an Hsd-family type I RM system. The homologous system of *H. pylori* J99-R3 appears to be functional but silenced, possibly due to the frame-shift in res preventing transcription or translation [10]. Our results may verify this hypothesis, as deletion of the HP0464 res had no effect on colonization, but repair of the frame-shift in HP0464 had a very pronounced effect, indicating that the system had been activated and that its function is detrimental to colonization. Interestingly, in 31 *H. pylori* genomes the status of gene HP0464 was found to be almost equally distributed between ON/OFF state (Additional file 1: Table S3). This observation suggests a metastable expression for gene HP0464.

**Figure 2:**
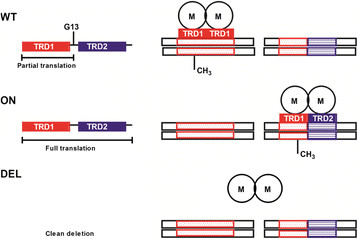
**Model for the function of phase variation in HP1471.** Expression of the target recognition domain TRD1 in OND79 leads to specific methylation of DNA sequence recognized by TRD1 (WT), indicated in red. In OND79 HP1471::ON the homopolymeric repeat (G13) is removed allowing expression of TRD1 and TRD2 resulting in specific methylation at DNA sequence recognized by TRD1/TRD2 (ON), indicated in red and blue. In OND79 ΔHP1471 neither TRD1 nor TRD2 are expressed leading to absence of methylation due to the non-functional methyltransferase (DEL).

Compared to WT both mutants (‘DEL’ and ‘ON’) of HP1471 had difficulty establishing colonization. The HP1471-HP1472 region is highly variable among *Helicobacter* strains. HP1471 encodes target recognition domains (TRDs) that determine DNA sequence specificity of the RM system and HP1472 encodes the mod. The homopolymeric repeat in HP1471 occurs directly between the two TRDs. We hypothesize that the TRDs can be considered as modules, with each module responsible for the recognition of a specific DNA sequence (Figure 2). The protein phase varies by alteration of the length of the homopolymeric repeat between the two modules, determining if only a single module or both modules of the protein are translated. In the wild-type strain only the first module is translated. In the ‘ON’ mutant both specificity modules of HP1471 are expressed and the specificity of the mod is altered to a different DNA motif. In the clean deletion mutant there is no DNA methylation. The fact that only WT and not the ‘DEL’ or ‘ON’ HP1471 mutants colonized mice suggests that expression of both target DNA recognition domains (TRD1 and TRD2) are required during colonization. This highlights a temporal role of phasevarions and DNA methylation in adaptation to the host. Movement of TRDs leading to differential genome methylation has been postulated to be a target for “epigenetic-driven adaptive evolution” [16]. Experimental evidence for a similar system, the type-I RM system encoded by JHP1422, has been obtained by Krebes *et al*. [10]. However, their analysis of HP1471/HP1472 could not detect methyltransferase activity in 26695 and could only detect activity in the homologous JHP1364/1365 when both TRDs of JHP1364 were expressed upon correction of the frame-shift [10]. These differences could reflect allelic diversity at this locus in different strains of *H. pylori*.

## 3 Conclusions

Despite the increasing understanding of the role of DNA methylation in host-pathogen interaction, we are unaware of any study where the *in vivo* significance of phasevarions to *H. pylori* has been investigated. Our results demonstrated that phase-variable methyltransferases are critical to *H. pylori* colonization, suggesting that genome methylation and generation of epigenetic diversity is important for colonization and pathogenesis of this gastric human pathogen. Our mutants fell into three categories; 1, those with little effect on colonization, 2, those where expression of the full-length protein was detrimental, 3, those where both mutations were detrimental. The last category suggests that differential genome methylation status of *H. pylori* cell populations, achieved by the phasevarion, is essential for host adaptation. Of note, the presence of the homopolymeric tract between the two DNA recognition domains TRD1 and TRD2 in HP1471-HP1472 RM system (Figure 2) is likely to change the specificity of the methyltransferase upon phase-variation and to alter *H. pylori* methylome. Thus differential genome methylation is one mechanism *H. pylori* may have evolved to colonize the host. Studies of phase-variable RM mutants falling in category 1 and 2, not strictly required for colonization, represent a future perspective to investigate the role of phasevarion in persistence of *H. pylori*.

## 4 Methods

Bacterial strains and primers used in this study are listed in the Additional file 1*. H. pylori* was routinely grown on Columbia blood agar (CBA) plates containing Columbia blood agar base (Oxoid) with 5% (v/v) horse blood and 5% (v/v) new-born calf serum supplemented with chloramphenicol (10 μg/ml) or streptomycin (10 μg/ml) where appropriate. Plates were incubated at 37°C for 24 – 48 h within sealed jars in a microaerobic atmosphere obtained using the Anoxomat™ MarkII system (Mart Microbiology B.V., The Netherlands) and the microaerophilic gas replacement protocol based on a gas mixture containing N_2_:H_2_:CO_2_ (85:5:10). *E. coli* DH10beta was used for routine cloning. *E. coli* strains were grown on Luria-Bertani broth with shaking (200 rpm), or on Luria-Bertani plates supplemented with ampicillin (100 μg/ml) or chloramphenicol (10 μg/ml) where appropriate at 37°C for 18 h.

Homologous genes to those of *H. pylori* 26695 were located within the unpublished genome sequence of OND79 (Ondek Pty. Ltd.) by BLAST (bl2seq). Gene sequences were annotated and homopolymeric tracts of greater than or equal to G7 or C7, and to A9 or T9 were located using Vector NTI Advance® 11.5.1 (Invitrogen).

Clean deletions of target genes were constructed using Xer-cise [17]. Briefly, the flanking regions of target genes were amplified by SOE-PCR [18] from OND79 genomic DNA with Phusion DNA polymerase (NEB) with primer pairs JG379/JG380 and JG381/JG382 for HP1369, JG385/386 and JG387/JG388 for HP1471, JG391/JG392 and JG393/JG394 for HP1522, JG397/JG398 and JG399/JG400 for HP0464, and JG403/404 and JG405/406 for HP0381. The resulting amplicons were digested with *EcoR*I (HP1369, HP1471, HP1522) or *Nco*I/*Sal*I (HP0464, HP0381) and ligated with the pGEMT fragment of pComB4-prep (pOND873) that had been digested with the same enzymes. The resulting vectors were digested with *BamH*I to allow the introduction of either a *BamH*I *rpsL-cat* fragment [19] between the flanking region of the target genes, generating pOND1467, pOND1468, pOND1469, pOND1470, and pOND1471 or the *BamH*I *difH-rpsL-cat-difH* fragment of pDifWT-RC, between the flanking region of the target genes, generating plasmids pOND1462, pOND1463, pOND1464, pOND1465, and pOND1466. OND79 was transformed with pOND1462, pOND1463, pOND1464, pOND1465, and pOND1466 by natural transformation and transformants were obtained on CBA plates containing 10 μg/ml chloramphenicol. Transformant colonies were patched onto CBA containing 10 μg/ml chloramphenicol prior to being transferred to CBA plates containing 10 μg/ml streptomycin. Genomic DNA was isolated from the clones and used as a template for PCR with primers JG402/JG407 for HP0381, JG378/JG383 for HP1369, JG384/389 for HP1471, JG390/JG395 for HP1522, and JG396/JG401 for HP0464 to screen for deletion of the target gene.

To construct mutants in which the homopolymeric tract had been replaced a strategy using replacement of the wild-type allele with *rpsL-cat* with a PCR amplicon encoding the mutant allele was pursued. OND79 was transformed with plasmid DNA of pOND1467, pOND1468, pOND1469, pOND1470, and pOND1471 by natural transformation and transformants were obtained on CBA containing 10 μg/ml chloramphenicol. Genomic DNA of transformant clones that were chloramphenicol sensitive and streptomycin resistant was used as a template for PCR to screen for replacement of the target gene with *rpsL-cat* with primers JG402/JG407 fzor HP0381, JG378/JG383 for HP1369, JG384/JG389 for HP1471, JG390/JG395 for HP1522, and JG396/JG401 SOE-PCR was used to amplify the target gene from OND79 genomic DNA using primers in which the homopolymeric tract sequence had been altered. Briefly, HP0381 was amplified with primer pairs JG403/409, JG410/JG412 and JG411/JG406, HP1369 was amplified with primer pairs JG379/JG414 and JG413/JG382, HP1471 was amplified with primer pairs JG385/JG416 and JG415/JG388, HP1522 was amplified with primer pairs JG391/JG418 and JG394/JG471, and HP0464 was amplified with primer pairs JG397/JG420 and JG400/JG419. Strains of OND79 in which the wild-type allele has been replaced with *rpsL-cat*[19] were transformed with the resulting amplicons and transformants were obtained on CBA containing 10 μg/ml streptomycin. Genomic DNA isolated from streptomycin resistant and chloramphenicol sensitive clones were used as a template to screen for replacement of *rpsL-cat* with the mutant allele with primers JG402/JG407 for HP0381, JG378/JG383 for HP1369, JG384/JG389 for HP1471, JG390/JG395 for HP1522, and JG396/JG401.

To perform colonization assays *Helicobacter* free C57BL/6 J mice were purchased from the Animal Resource Centre (Perth, Western Australia). Studies were performed with approval from the UWA Animal Ethics Committee (approval no. 07/100/598). Each eight-week-old mouse was orogastrically inoculated with approximately 10^9^ CFUs of *H. pylori* harvested from an overnight agar plate culture into BHI broth. Colonisation of mice inoculated with OND79 wild-type or mutant strains was evaluated 2 weeks after challenge as described [20].

## Competing interests

JCG, HN, AF, BJM and MB report grants from Ondek Pty Ltd, grants from Vice-Chancellor's Fund at The University of Western Australia, a NHMRC Sir McFarlane Burnett Fellowship grant (572723) to BJM and ownership of Ondek shares/share options during the conduct of the study; JCG, HN, AF and MB are employees of Ondek Pty Ltd.

## Authors’ contributions

JCG, study concept and design; acquisition of data; analysis and interpretation of data; drafting of the manuscript; HN, critical revision of the manuscript for important intellectual content; technical support; AF, animal ethics and experimentation; BJM, study critical revision of the manuscript for important intellectual content; obtained funding; MB, study concept and design; analysis and interpretation of data; drafting of the manuscript; critical revision of the manuscript for important intellectual content; obtained funding; study supervision. All authors read and approved the final manuscript.

## Additional file

## Supplementary Material

Additional file 1: Table S1. Bacterial strains and plasmids used in this study. **Table S2.** Oligonucleotides used in this study. **Table S3.** HP0464 on/off status in 31 *H. pylori* strains. Click here for file
