# Supplementary material for: Phase-variable restriction/modification systems are required for Helicobacter pylori colonization
Source: Gut Pathog. 2014 Sep 5;6:35. doi: 10.1186/s13099-014-0035-z (PMC4209511; doi:10.1186/s13099-014-0035-z)
Supplement: Additional file 1: Table S1. — Bacterial strains and plasmids used in this study. Table S2. Oligonucleotides used in this study. Table S3. HP0464 on/off status in 31 H. pylori strains. [file s13099-014-0035-z-S1.docx]

**Additional file 1**

**Table S1.** Bacterial strains and plasmids used in this study.

| **Strain or plasmid** | **Genotype/description** | **Source** |
| --- | --- | --- |
|  |  |  |
| ***H. pylori* strains** |  |  |
| OND79 | Wild-type strain, Strp^R^ | This study |
| OND3684-3686 | OND79 *ΔHP1522* – three independent clones | This study |
| OND3687-3689 | OND79 *ΔHP0464* – three independent clones | This study |
| OND3670-3672 | OND79 *ΔHP0381* – three independent clones | This study |
| OND3885-3887 | OND79 *ΔHP1471* – three independent clones | This study |
| OND3883, 3884 | OND79 *ΔHP1369* – two independent clones | This study |
| OND3891-3893 | OND79 *HP1369::rpsL-cat* – three independent clones | This study |
| OND3894-3896 | OND79 *HP1471::rpsL-cat* – three independent clones | This study |
| OND3897-3899 | OND79 *HP1522::rpsL-cat* – three independent clones | This study |
| OND3900-3902 | OND79 *HP0464::rpsL-cat* – three independent clones | This study |
| OND3913-3915 | OND79 *HP0381::rpsL-cat* – three independent clones | This study |
| OND3903-3905 | OND79 *HP1369::ON* – three independent clones | This study |
| OND3906 | OND79 *HP0464::ON* | This study |
| OND3907-3909 | OND79 *HP1471::ON* – three independent clones | This study |
| OND3910-3913 | OND79 *HP1522::ON* – three independent clones | This study |
| OND3958-3960 | OND79 *HP0381::ON* – three independent clones | This study |
|  |  |  |
| ***E. coli* strains** |  |  |
| DH10β | *F^-^ endA1 recA1 galE15 galK16 nupG rpsL ΔlacX74 Φ80lacZΔM15 araD139 Δ(ara,leu)7697 mcrA Δ(mrr-hsdRMS-mcrBC) λ^-^* | Life technologies |
|  |  |  |
| **Plasmids** |  |  |
| pComB4-prep | pGEMT containing flanking regions of *comB4* – pOND873 | [[16](#_ENREF_16)] |
| pDifWT-RC | Vector containing *difH-rpsL-cat-difH* cassette – pOND849 | [[20](#_ENREF_20)] |
| pOND1462 | pGEMT containing flanking regions of *HP1522* with internal *difH-rpsL-cat-difH* cassette | This study |
| pOND1463 | pGEMT containing flanking regions of *HP0464* with internal *difH-rpsL-cat-difH* cassette | This study |
| pOND1464 | pGEMT containing flanking regions of *HP0381* with internal *difH-rpsL-cat-difH* cassette | This study |
| pOND1465 | pGEMT containing flanking regions of *HP0464* with internal *difH-rpsL-cat-difH* cassette | This study |
| pOND1466 | pGEMT containing flanking regions of *HP1471* with internal *difH-rpsL-cat-difH* cassette | This study |
| pOND1467 | pGEMT containing flanking regions of *HP1369* with internal *rpsL-cat* cassette | This study |
| pOND1468 | pGEMT containing flanking regions of *HP1471* with internal *rpsL-cat* cassette | This study |
| pOND1469 | pGEMT containing flanking regions of *HP1522* with internal *rpsL-cat* cassette | This study |
| pOND1470 | pGEMT containing flanking regions of *HP0464* with internal *rpsL-cat* cassette | This study |
| pOND1471 | pGEMT containing flanking regions of *HP0381* with internal *rpsL-cat* cassette | This study |

**Table S2.** Oligonucleotides used in this study

| **Name** | **Sequence** |
| --- | --- |
| JG378 | tggcactttcagggatattag |
| JG379 | aaatttGAATTCgaaatgagagcgtttaaagcg |
| JG380 | cttaccaGGATCCcgctttaggggtaggatctaa |
| JG381 | ctaaagcgGGATCCtggtaaggagaaaatcatggc |
| JG382 | AAATTTgaattcCATGCCTAAAAGCAATAACGC |
| JG383 | AGGGTCGTAAAAATAGCCTTC |
| JG384 | aaacgcgcaaaaacccttgcg |
| JG385 | aaatttGAATTCgtggcgctttccaaaacaatc |
| JG386 | CTCAACggatccTGATCTTATGGTAAGCAATACCGC |
| JG387 | gatcaGGATCCgttgagttggctattaagggg |
| JG388 | AAATTTgaattcTCATGGGCGAACTTTATAGCG |
| JG389 | AGCCTGGATAAAGCCATAAGC |
| JG390 | catcacttcttgttcaccctc |
| JG391 | aaatttGAATTCagcgctgatttgagttaccac |
| JG392 | GGGTAAggatccTAGGGGGTAGAGGTGAAAATC |
| JG393 | cccctaGGATCCttacccaattccaccttaattttc |
| JG394 | cgatttagatgaattcgctttag |
| JG395 | gttacaagaagcgatccaatc |
| JG396 | gcgtgtaaatgggcattttgc |
| JG397 | aaatttCCATGGgcatgctcgttggaaaaatcc |
| JG398 | gtgttcGGATCCtaaGAAACCACCCTAAAAACTGCC |
| JG399 | GTTTCTTAGGATCCgaacacccctttgatttaagg |
| JG400 | AAATTTGTCGACttggtaatgggccaagtttag |
| JG401 | TACTGGGATTAAAGAACACGC |
| JG402 | gaagaagcgggaaattatgcc |
| JG403 | aaatttCCATGGaaacctttctgaaacgctagg |
| JG404 | gaatgatggatccCTTTGTTTAGGGCTTGTGAaAG |
| JG405 | caaagGGATCCatcattcttttagccgctgag |
| JG406 | aaatttGTCGACaagctctaaatcaatgccgtc |
| JG407 | attctttgatcctcccacgac |
| JG409 | GTTTTAGaGGtGGaTTAGAGTCTGAAATTTTATTAGGC |
| JG410 | CTAAtCCaCCtCTAAAACCTTTTTGCGATAATCC |
| JG411 | CTTTTTGGtGGaGTTAAAGGCGATGAAATCTTAAAAG |
| JG412 | CTTTAACtCCaCCAAAAAGGGCTTCGTGCGGTTC |
| JG413 | CTTTAATGGaGGaGGTGATAACTTTGTAGCGAATATTAG |
| JG414 | GTTATCACCtCCtCCATTAAAGACTTCGTCCATGAGC |
| JG415 | GTATTaCCtCCaCCtCCAGAATTATTGAAAACATTAAGG |
| JG416 | CAATAATTCTGGaGGtGGaGGtAATACCCCATGCGGCTTAACATGG |
| JG417 | CATTAAATAACCCtCCaCCTCCACTTAATTTTTGATAATCGC |
| JG418 | GGAGGtGGaGGGTTATTTAATGGCTTGAACGCC |
| JG419 | CATTTTCtGGaGGtGTAATATCTAGCTTATGAGCTTCAAC |
| JG420 | GATATTACaCCtCCaGAAAATGAACAAAATTATCAAAATC |

**Table S3.** HP0464 on/off status in 31 *H. pylori* strains

| Strain | Status | HP464 sequence bp 610 to 633 (strain 26695) | | |
| --- | --- | --- | --- | --- |
| 26695 | on | ATTACCCCCCCCCCCCCCCAAAAA | |  |
| Rif2 | on | ATTACCCCCCCCCCCCCCCAAAAA | |  |
| Rif1 | on | ATTACCCCCCCCCCCCCCCAAAAA | |  |
| G27 | off | ATTACCCCCCCCCCCCCCC--GAAAA | |  |
| j166 | off | ATTACCCCCCCCCCCCC----GA | |  |
| oki422 | off | ATTACCCCCCCCCCCCCCC--GAA | |  |
| oki898 | off | ATTACCCCCCCCCCCCCCGAAAATGATCA | | |
| oki112 | off | ATTACCCCCCCCCCCC-----GAA | |  |
| oki102 | off | ATTACCCCCCCCC--------GAA |  |  |
| B8 | off | ATTACCCCCCCCCC-------GAA | |  |
| BMO13B | on | ATTACCCCCCCCCCCCCC---GAA | |  |
| BMO13A | on | ATTACCCCCCCCCCCCCC---GAA | |  |
| HPAG1 | on | ATTACCCCCCCCCCCCCC---GAA | |  |
| India7 | on | ATTACCCCCCCCCCC------GAA | |  |
| XZ274 | off | ATTACCCCCCCCCCC----AAAAA | |  |
| Sat464 | on | ATTACCCCCCCCCCCCGAAAAAACCATCAA | | |
| SNT49 | on | ATTACCCCCCCCCCCCCAGAAAATGATCAA | | |
| Lithuania 75 | on | ATTACCCCCCCCCCCCC----------G---AAAA | | |
| ELS37 | off | ATTACCCCCCCCCCCC-----GA | |  |
| B38 | off | ATTACCCCCCCCCCCCCCC--GAA | |  |
| NY40 | on | ATTACCCCCCCCCCCCCC---GAA | |  |
| UM037 | off | ATTA----CCCCCCCCCCCGAAAA | |  |
| Cuz20 | off | ATTACCCCCCCCCCCCC----GAA | |  |
| 35A | off | ATTACCCCCCCCCCCCC----GAA | |  |
| Puno 120 | on | ATTACCCCCCCCCCCC---GAA | |  |
| oki673 | off | ATTACCCCCCCCCCCCCC-GAA | |  |
| v225d | off | ATTACCCCCCCCCC-----GAA |  |  |
| Shi 112 | off | ATTACCCCCCCCCCCC-----GAA | |  |
| UM066 | off | ATTACCCCCCCCCCCC-----GAA | |  |
| Shi 169 | on | ATTACCCCCCCCCCC------------GA-A | |  |
| Shi 470 | on | ATTACCCCCCCCCCCCCC---------GA--A | | |
